# Anthocyanin and proanthocyanidin from *Aronia melanocarpa* (Michx.) Ell.: Purification, fractionation, and enzyme inhibition

**DOI:** 10.1002/fsn3.3377

**Published:** 2023-05-04

**Authors:** Limei Chen, Wuxi Chen, Demao Li, Xiumin Liu

**Affiliations:** ^1^ Tianjin Key Laboratory for Industrial BioSystems and Bioprocessing Engineering Tianjin Institute of Industrial Biotechnology, Chinese Academy of Sciences Tianjin China; ^2^ National Innovation Centre for Synthetic Biology Tianjin China; ^3^ Hebei Jiaotong Vocational and Technical College Hebei Shijiazhuang China

**Keywords:** anthocyanins, *Aronia melanocarpa* (Michx.) Ell., lipase, proanthocyanidins, α‐amylase, α‐glucosidase

## Abstract

*Aronia melanocarpa* (Michx.) Ell. is a rich source of anthocyanins and proanthocyanidins with confirmed health benefits. Individual cyanidin glucosides (cyanidin 3‐galactoside, cyanidin 3‐arabinoside, cyanidin 3‐xyloside, and cyanidin 3‐glucoside) of anthocyanins (calculated by individual cyanin glycoside fractions was 419.9 mg/100 g FW) were isolated by Sephadex LH‐20 column and different parts of proanthocyanidins with a different mean degree of polymerization (mDP) were fractionated by the solubility differences in different solvents. The composition of different mDP of proanthocyanidins was as follows: monomers (1.51%), oligomer (mDP of 4.2 ± 0.9, 20.57%), CPP‐50 (mDP of 78.9 ± 4.1, 22.17%), CPP‐60 (mDP of 66.1 ± 1.2, 27.94%), CPP‐70 (mDP of 36.8 ± 3.9, 36.8%), CPP‐75 (mDP of 25.2 ± 1.3, 6.14%), CPP‐L (mDP of 10.2 ± 2.6, 6.95%), and there were recycling loss of 0.34%. Cyanidin 3‐glucoside showed the strongest inhibition effects on α‐amylase and lipase and cyanidin 3‐arabinoside showed the strongest inhibition effect on α‐glucosidase, while cyanidin 3‐xyloside has no inhibition effect on the α‐amylase, and cyanidin 3‐galactoside, cyanidin 3‐arabinoside, and cyanidin 3‐xyloside have no inhibition effects on lipase. The inhibition effect of proanthocyanidins with different mDP to the enzymes all showed high negative correlations between the mDP and IC_50_ (half‐maximal inhibitory concentration). This study suggests that *A. melanocarpa* (Michx.) Ell. can have beneficial effects due to inhibition of the digestion enzyme.

## Graphical abstract

The content, composition and enzyme inhibition activity of anthocyanin and proan‐thocyanidins of *A. melanocarpa* (Michx.) Ell. from China were similar to those in the United States and Europe. Anthocyanin and proanthocyanidins inhibited the activity of digestion enzyme, which is one of the important physiological functions of *A. melanocarpa* (Michx.) Ell.

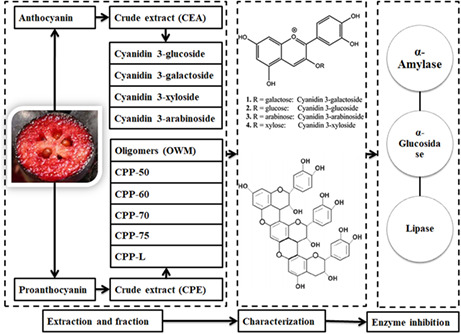

## 1 INTRODUCTION

With the increase in people's income and living standard, consumers put forward higher requirements for the health, nutrition, safety, and function of food, resulting in the continuous growth of market demand for healthy food with biologically active compounds. It has received wide attention that berries with high content of bioactive substances such as different kinds of polyphenols (anthocyanins and proanthocyanidins), organic acid, and polysaccharides are associated with a lower risk of arteriosclerosis, anti‐diabetic effects, obesity (Gironés‐Vilaplana et al., 2014), etc.

Among the functional berries, greatly increased research interests have been put on *A. melanocarpa* (Michx.) Ell. with a high content of polyphenols (including anthocyanins, flavonols, flavanols, proanthocyanidins, phenolic acids, etc.) (Sidor et al., 2019) and possess one of the highest antioxidant activities among plant species (Denev et al., 2012). Anthocyanins are mainly composed of cyanidin 3‐glucoside, 3‐galactoside, 3‐xyloside, and 3‐arabinoside, which are the main source of black color (Veberic et al., 2015). Flavonols present in *A. melanocarpa* (Michx.) Ell. belong to a diverse group of compounds, which mainly consist of quercetin derivatives (quercetin‐3‐glucoside, 3‐galactoside, 3‐rutinoside, 3‐robinobioside, and 3‐vicianoside), isorhamnetin 3‐galactoside, 3‐glucoside, 3‐neohesperidoside, 3‐rutinoside, myricetin and kaempherol 3‐galactoside, and 3‐glucoside. Proanthocyanidin of *A. melanocarpa* (Michx.) Ell. is mainly composed of (−)‐epicatechin and trace amounts of (+)‐catechin with different mean degree of polymerization (mDP). *A. melanocarpa* (Michx.) Ell. also contain phenolic acids, among which dominating are chlorogenic and neochlorogenic acids (Sidor et al., 2019). These bioactive compounds contributing to the antioxidative potential and alleviation of the related diseases were confirmed in vitro, in vivo, and clinically. They were reported to have the effects of regulating the expression of genes critical for intestinal cholesterol flux in Caco‐2 cells, antiatherogenic, cardioprotective, gastroprotective, antioxidant, anti‐inflammation, anti‐aging, anti‐cancer, etc. (Bräunlich et al., 2013; Sosnowska et al., 2018). Thus, generous consumption of *A. melanocarpa* (Michx.) Ell. is recommended in dietary guidelines worldwide (Vázquez‐Espinosa et al., 2019). However, due to the bitterness or astringency taste, they are not usually consumed as fresh fruits, but processed as jams, jellies, fruit syrups, juice, energy drinks (Jurikova et al., 2017), or concentrated extracts (Jurgoński et al., 2008).

Extraction is the crucial step for isolation, identification, and use of phenolic compounds. However, there is no general extraction method, and different methods will be used according to different raw materials. Pretreatment may be a good choice to release the polyphenol compounds, such as fermentation, enzyme lysis, grinding, and freeze‐thawing. The most commonly used techniques for the isolation of phenolic compounds are solvent extraction (with or without assistance of microwave and ultrasound) and supercritical fluid extraction. This method has the advantages of being a simple process, low cost, and high purity. Different adsorbents were tested, such as Amberlite XAD7HP absorption and acidified ethanol elution (Galván D'Alessandro et al., 2013), Sep‐Pak C‐18 cartridges absorption, 1% acetic acid acidified water (Jing et al., 2008) or methanol (Sosnowska et al., 2018) elution for anthocyanin, and Sephadex LH‐20 for the purification of anthocyanins (Bräunlich et al., 2013) and proanthocyanidins (Fan et al., 2007) in order to remove the concomitant substances such as sugars, amino acids, and proteins from anthocyanin and/or proanthocyanidin‐containing extracts. For the fractionation of different mDP of proanthocyanidins, a rapid method based on liquid/liquid extraction and relative solubility of these compounds in different solvents (water, ethyl acetate, methanol, and chloroform) was established (Saucier et al., 2001).

The prevalence of diabetes and obesity is alarmingly increasing in the last few decades, leading to many serious public health concerns worldwide. α‐Glucosidase, α‐amylase, and lipase are the key enzymes that affect the digestion and absorption of major carbohydrates and lipids in diet (Türkan et al., 2020). Thus, inhibiting α‐glucosidase, α‐amylase, and pancreatic lipase would prevent the breakdown of carbohydrates and triglyceride and delay the absorption of glucose and fatty acids into the systemic circulation and adipocytes (Rajan et al., 2020). However, the conventional α‐glucosidase, α‐amylase, and lipase inhibitor available in clinics are all chemicals (side effects often occurred), and identifying safe clinical alternatives from plants to inhibit these enzymes have been considered a significant advancement (Rajan et al., 2020).

**FIGURE 1:**
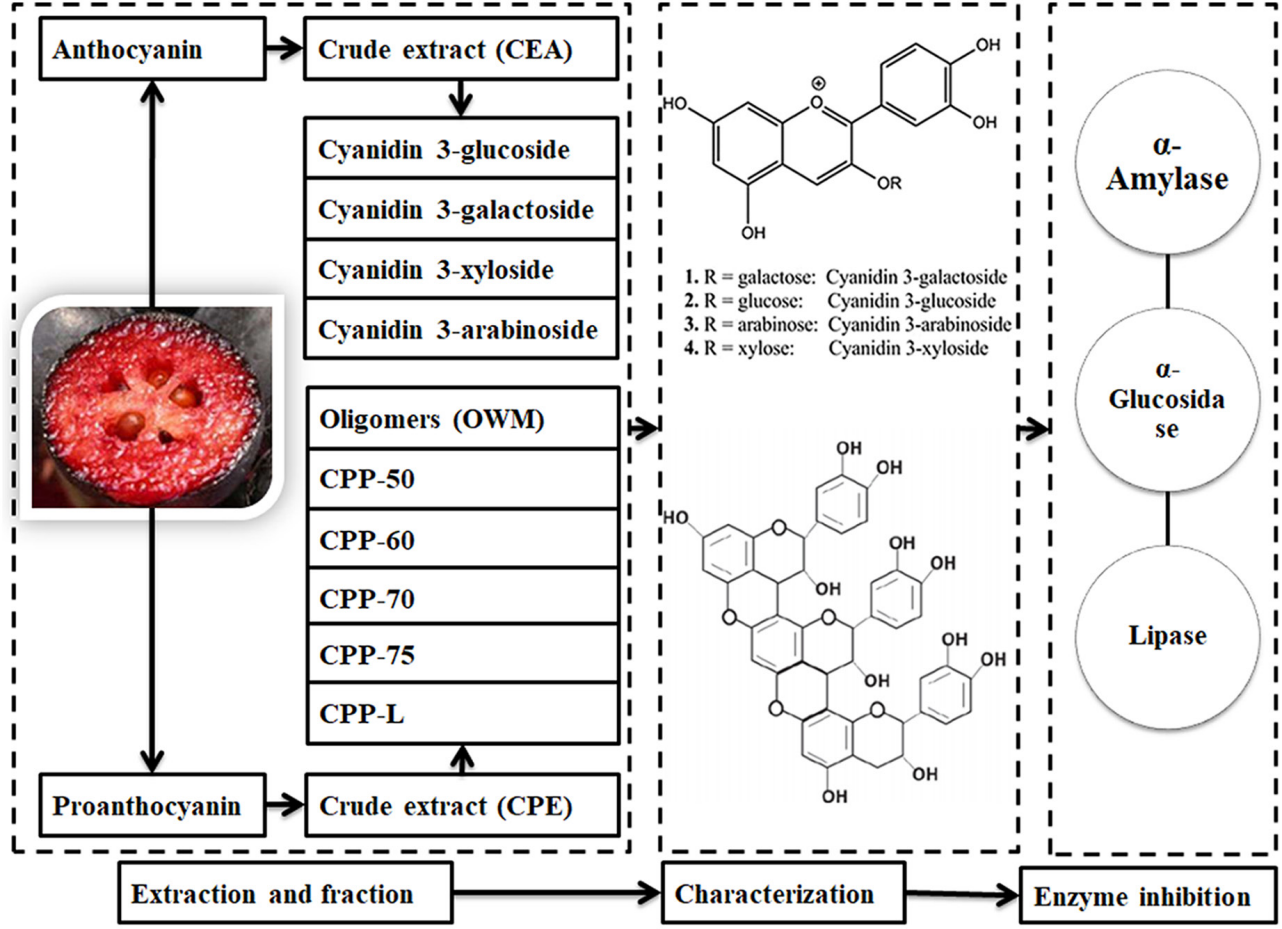
Flow chart of extraction, fractionation, characterization, and enzyme inhibition.

Reports indicated that phenolic in *Aronia* can inactivate α‐amylase, α‐glucosidase, and lipase through non‐specific binding to enzymes (Bräunlich et al., 2013). However, crude polyphenol was studied and the individual effects of pure chemicals have not been considered, which will be important for the studying of the mechanism of the inhibition. Therefore, the individual cyanidin glycosides of anthocyanins and proanthocyanidins with different mDP were fractionated and the enzyme inhibition effects were studied (Figure 1). This study will be useful to elucidate the inhibition effects of polyphenol from *A. melanocarpa* (Michx.) Ell. for obesity and diabetes and helpful for product development.

## 2 MATERIALS AND METHODS

### 2.1 Chemicals and materials

All the chemicals used for analyses were of analytical grade and high‐performance liquid chromatography (HPLC) chemicals were of HPLC purity and were all purchased from Sinopharm Chemical Reagent Co., Ltd (Shanghai, China). Lipase from human porcine pancreas (EC 3.1.1.3), α‐amylase from human pancreas (EC 3.2.1.1), and α‐glucosidase from *Bacillus stearothermophilus* (EC 3.2.1.20) were obtained from Sigma‐Aldrich, Inc (Shanghai, China). Standards of cyanidin 3‐galactoside, cyanidin 3‐arabinoside, cyanidin 3‐xyloside, cyanidin 3‐glucoside, catechin, and epicatechin were obtained from Sigma‐Aldrich, Inc. (Shanghai, China). Amberlite XAD7HP, Sephadex LH‐20, and ENVI 18 DSK SPE were obtained from Sigma‐Aldrich, Inc (Shanghai, China).

*Aronia melanocarpa* (Michx.) Ell. were manually harvested at technological maturity by Liaoning Fukangyuan Black Chokeberry Technology Co., Ltd. (September 18, 2018) and was freeze stored until use.

### 2.2 Isolation of anthocyanins from *A. melanocarpa* (Michx.) Ell.

The pre‐grounded slurry of *A. melanocarpa* (Michx.) Ell. (4 kg) was subjected to n‐hexane (6:1, v/m) to remove lipophilic substances, after which was divided into two parts (part 1 for anthocyanins isolation and the other part for proanthocyanidins extraction) as pre‐treated slurry. Then, the slurry was extracted twice by ethanol solution (60% ethanol with 0.1% HCl, v/v) for 2 h at ambient temperature bubbled with nitrogen, and was centrifuged at 9090 *g* for 6 min and the supernatant was collected and combined. Then, the ethanol was removed from the supernatant and the retained solution was concentrated through rotatory evaporation under vacuum at 40°C to give the crude extract of anthocyanins (CEA). The CEA was subjected to a bed of Amberlite XAD‐7HP (5 × 50 cm column) until the eluate has no more anthocyanins detected by pH differential method (Jing et al., 2008), followed by elution with methanol (0.1% HCl) to give the anthocyanin‐enriched extract (AEE). The AEE was then purified by a Sephadex LH‐20 column (5 × 150 cm) by a step gradient of 15% and 30% methanol (0.1% HCl v/v), the fractionation steps follow Bräunlich et al. (2013), and was detected with HPLC at 520 and 280 nm for purity. Then, the individual of the cyanidin glycosides (cyanidin 3‐galactoside, cyanidin 3‐arabinoside, cyanidin 3‐xyloside, and cyanidin 3‐glucoside) were obtained.

### 2.3 Fractionation of proanthocyanidins from *A. melanocarpa* (Michx.) Ell.

#### 2.3.1 Crude proanthocyanidins extract

The pre‐treated slurry of *A. melanocarpa* (Michx.) Ell. was extracted two times successively with 7 L of acetone:water (70:30, v/v) bubbled with nitrogen with mechanical agitation for 6 h. All the slurries were centrifuged at 8000 rpm for 6 min and the supernatants were combined and evaporated at 40°C using a vacuum rotary evaporator to remove acetone. And the aqueous solution was freeze‐dried to give the crude proanthocyanidins extract (CPE) powder.

#### 2.3.2 Isolation of crude oligomer fraction (COF) and crude polymerized proanthocyanidins (CPP)from CPE

The CPE powder was dissolved in redistilled water to 5 g/L followed by ethyl acetate extraction two times with 1:1 (v/v) of the organic and aqueous phase. The ethyl acetate extract was evaporated at 40°C using a vacuum rotary evaporator to dry for crude oligomer fraction from CPE (COF). And the CPP was retained in the aqueous followed by freeze drying for the powder of CPP.

#### 2.3.3 Removal of monomer from COF

The COF powder was dissolved to 150 mL by redistilled water. Then solid‐phase extraction (SPE) with column (ENVI 18, Supelco) was conducted by applying the dissolved COF on each column. The monomers were then removed by eluting diethyl ether until no monomers (epicatechin) were detected in the eluent by HPLC (Saito et al., 2006). Then, the remained oligomers were eluted with methanol until no more oligomers were detected by Folin–Ciocalteu method (Tan et al., 2017). Then, the methanol was evaporated, the residue was dissolved in minimum water, and freeze‐dried to obtain oligomers without monomers (OWM).

#### 2.3.4 Fractionation of the polymer with different mDP from CPP

CPP powder was prepared by freeze drying the aqueous solution, followed by dissolving it with methanol. The same volume of chloroform (1:1, v/v) was added to the CPP–methanol solution. Then, the precipitate was harvested by filtration and recovered by methanol washing. Then, this methanol solution was evaporated and the residue was dissolved in water and freeze‐dried as a fraction of CPP fractionated by 50% chloroform (CPP‐50). The retained filtrate was repeatedly precipitated by adding more chloroform successively to the volume ratio of 1.5:1, 2.33:1, and 3:1 (v/v, chloroform:methanol), and the corresponding precipitate was harvested by filtration, washed in methanol, dissolved in water, and freeze‐dried to make the fraction of CPP‐60, CPP‐70, and CPP‐75. And the last filtrate was also evaporated, redissolved in water, and freeze‐dried to make CPP‐L fraction. All these samples were kept at −80°C until further studies (Saucier et al., 2001; Sosnowska et al., 2018).

### 2.4 Thiolysis of partially purified proanthocyanidins

Different kinds of partially purified *A. melanocarpa* (Michx.) Ell. proanthocyanidins (10 mg) were dissolved in 1.0 mL of 95% ethanol to prepare a 10 mg/mL proanthocyanidins solution. The thiolysis method was carried out according to Gao et al. (2018).

### 2.5 Determination of total phenolic contents

The total phenolic content was measured by the Folin–Ciocalteu method after adjustments (Vázquez‐Espinosa et al., 2019) with 60% ethanol (with 0.1% HCl) *A. melanocarpa* (Michx.) Ell. extracts. Gallic acid was used as a calibration standard, and results were expressed as gallic acid equivalents (GAE) per 100 g of fresh weight (FW) (mg of GAE/100 g FW).

### 2.6 Determination of total anthocyanins

Total anthocyanin contents of *A. melanocarpa* (Michx.) Ell. extracts were measured using the pH differential method (Lee et al., 2005) with 60% ethanol (with 0.1% HCl). Results are expressed as milligrams of cyanidin 3‐glucoside equivalents per 100 g of fresh weight (mg of cyanidin 3‐glucoside/100 g FW).

### 2.7 Determination of total proanthocyanidins

Proanthocyanidins were depolymerized into anthocyanidins by use of an n‐BuOH–HCl–ferric ammonium sulfate mixture and HPLC was used to determine the content as stated in Skupien and Oszmianski (2007). (−) Epicatechin was used as a calibration standard and results were expressed as mg (−)epicatechin equivalents (EE) per 100 g of FW (mg of EE/100 g FW).

### 2.8 HPLC and mass spectra determination of the cyanidin glycosides, proanthocyanidins, and thiolytic products

An Agilent 1260 Infinity HPLC (Agilent Technologies, Santa Clara, CA, USA) was used for the purity check of the cyanidin glycosides, (−)epicatechin, catechin, and thiolysis products by an Eclipse XDB‐C8 (4.6 × 150 mm, 5 μm) column (Agilent Technologies, Santa Clara, CA, USA) according to the method of Bräunlich et al. (2013) with a diode array detector (DAD). Anthocyanin standard stock solutions were prepared in methanol containing 0.1% formic acid and were used to identify the compounds. Wavelengths of (−)epicatechin at 280 nm and anthocyanin glycosides at 520 nm were determined. The mass spectra of thiolytic products were acquired in positive mode using electrospray ionization on an Agilent 6220 ESI‐TOF mass spectrometer. The mDP was calculated by the following formula (Ci et al., 2018):

(1) $$\mathrm{mDP}=\left[1+\left(\frac{\text{area of catechin and epicatechin derivatives}}{\text{area of catechin and epicatechin}}\right)\right]$$

### 2.9 Enzyme inhibition essays

#### 2.9.1 α‐Amylase inhibition studies

α‐Amylase inhibitory activity was determined using the method from Tan et al. (2017), with a slight modification as Table 1 indicates. One hundred microliters of different concentrations of the fractionated compounds and 100 μL of the enzyme solution (5 mg/L) were mixed in a centrifuge tube at ambient temperature for 10 min. After adding 200 μL, 0.5% soluble starch with phosphate solution as a buffer (25 mmol/L, pH = 6.8), the tube was incubated at 37°C for 5 min. Then, 1 mL of DNS color reagent solution (96 mM 3,5‐dinitrosalicylic acid and 5.31 M sodium potassium tartrate in 2 M NaOH) was added into the tube. The tube was placed into a boiling water bath for 5 min to inactivate the enzyme. Then, the solution was diluted by adding 3 mL of distilled water. Then, 200 μL of the mixture was taken and added to a 96‐well plate and the absorption at 540 nm was determined. To eliminate the background absorbance produced by the fractionated compounds, an appropriate extract control without enzyme was included. α‐Amylase inhibitory activity was measured at five different concentrations, and a logarithmic regression curve was established to calculate IC_50_ values (mg/mL) (Tan et al., 2017). The α‐amylase inhibitory activity was expressed as function 2 and the As, Ab, At, and Ac indicated the absorbance in different solutions as shown in Table 1. The inhibitory activity was measured at five different concentrations, and a logarithmic regression curve was established to calculate IC_50_ values.

(2) $$\alpha -\text{Amylase inhibitory activity}\left(\%\right)=\left[1-\left(\frac{\mathrm{As}-\mathrm{Ab}}{\mathrm{At}-\mathrm{Ac}}\right)\right]\times 100\%$$

**TABLE 1:** The mixture solution for α‐amylase inhibitory activity determination.

Treatments	Fractionated compounds	Starch solution	α‐Amylase	DNS solution	A_540nm_
Samples	√	√	√	√	As
Enzyme blank	√	√	X	√	Ab
Sample blank	B	√	√	√	At
Blank	B	√	X	√	Ac

*Note*: √ Indicates that included in the reaction mixture; X indicates that not included in the reaction mixture; and B indicates only buffer.

#### 2.9.2 α‐Glucosidase inhibition studies

α‐Glucosidase inhibitory activity was determined according to Gong et al. (2020) and Tan et al. (2017) with modifications (Table 2). Ten μL of each fractionated compound with appropriate concentration were mixed with 20 μL of 2.4 mM 4‐nitrophenyl‐β‐D‐glucuronide (pNPG) solution (dissolved in 0.1 M, pH 6.8 phosphate buffer), and 10 μL of 3 U/mL enzyme solution was added into a 96‐well plate to start the reaction at 37°C for 10 min. Then, 40 μL of Na_2_CO_3_ (1 mol/L) was added to the reaction mixture to terminate the reaction followed by determining the absorbance at 405 nm by a microtiter plate reader. The percentage of inhibition was calculated using Equation 3. α‐Glucosidase inhibitory activity was measured at five different concentrations, and a logarithmic regression curve was established to calculate IC_50_ values (Gong et al., 2020; Tan et al., 2017).

(3) $$\alpha -\text{Glucosidase inhibitory activity}\left(\%\right)=\left[1-\left(\frac{\mathrm{As}-\mathrm{Ab}}{\mathrm{At}-\mathrm{Ac}}\right)\right]\times 100\%$$

**TABLE 2:** The mixture solution for α‐ glucosidase inhibitory activity determination.

Treatments	Fractionated compounds	pNPG solution	α‐Glucosidase	A_540nm_
Samples	√	√	√	As
Enzyme blank	√	√	X	Ab
Sample blank	B	√	√	At
Blank	B	√	X	Ac

*Note*: √ Indicates that included in the reaction mixture; X indicates that not included in the reaction mixture; and B indicates only buffer.

#### 2.9.3 Lipase inhibition assay

The lipase inhibitory activity was determined according to the method described by Tan et al. (2017) with modifications (Table 3). Three hundred and fifty μL phosphate buffer (0.05 M, pH 7.6), 150 μL of porcine lipase enzyme solution (50 mg/mL), and 50 μL of fractionated compounds were added in a centrifuge tube (1.5 mL size) and incubated at 37°C for exactly 10 min. Then, 450 μL pNP laurate substrate was added to the reaction mixture and blended, followed by incubation at 37°C for 120 min without light. The reaction mixture was centrifuged for 10 min at 8000 ***g*** and the supernatant of 200 μL was taken into a 96‐well plate to determine the absorbance at 405 nm. The percentage inhibition was calculated using Equation 4. Lipase inhibitory activity was measured at five different concentrations, and a logarithmic regression curve was established to calculate IC_50_ values (Tan et al., 2017).

(4) $$\text{Lipase inhibitory activity}\left(\%\right)=\left[1-\left(\frac{\mathrm{As}-\mathrm{Ab}}{\mathrm{At}-\mathrm{Ac}}\right)\right]\times 100\%$$

**TABLE 3:** The mixture solution with different compositions for lipase inhibitory activity determination.

Treatments	Fractionated compounds	pNPP solution	Lipase	A_405nm_
Samples	√	√	√	As
Enzyme blank	√	√	X	Ab
Sample blank	B	√	√	At
Blank	B	√	X	Ac

*Note*: √ Indicates that included in the reaction mixture; X indicates that not included in the reaction mixture; and B indicates only buffer.

### 2.10 Statistics

All measurements were performed in triplicate and the results were presented as mean ± standard deviation (SD). The data and figures were processed with Origin 9.0 and Adobe Photoshop CC 2018. And SPSS 26.0 was used to evaluate whether the data of mDP and IC_50_ of different enzymes conform to normal distribution.

## 3 RESULTS AND DISCUSSION

### 3.1 Characterization of the *A. melanocarpa* (Michx.) Ell. Fruits

The contents of total polyphenol, proanthocyanidins, and anthocyanin of *A. melanocarpa* (Michx.) Ell. are indicated in Table 4 and were also compared with several studies. It indicates that *A. melanocarpa* (Michx.) Ell. also is a good source of phenol, anthocyanin, and proanthocyanidins planted in China. However, different sources of the *Aronia* fruits showed different contents of these compounds, which probably depend on variety, cultivation conditions, and harvest date (Kokotkiewicz et al., 2010). Proanthocyanidins accounted for 42.20%, while anthocyanins contributed only 17.28% of the total phenolic content for *A. melanocarpa* (Michx.) Ell.

**TABLE 4:** Content of total polyphenol, proanthocyanidins, and anthocyanin of *A. melanocarpa* (Michx.) Ell.

	Total polyphenol (mg of GAE/100 g FW)	Total proanthocyanidins (mg of EE/100 g FW)	Total anthocyanin (mg of cyanidin 3‐glucoside/100 g FW)	References
*A. melanocarpa* (Michx.) Ell.	3414.1 ± 12.2	1440.7 ± 15.1	590.0 ± 13.6	This study
*A. prunifolia*	2996.0 ± 172	4790.0	497.0 ± 20	Wangensteen et al. (2014)
*A. melanocarpa*	2556.0	–	429.0	Zheng and Wang (2003)
*A. melanocarpa*	2010.0	663.7	1480.0	Wu et al. (2004)

### 3.2 Extraction, fractionation, and characterization of the phenolic compounds

#### 3.2.1 Extraction, fractionation, and characterization of anthocyanins

**FIGURE 2:**
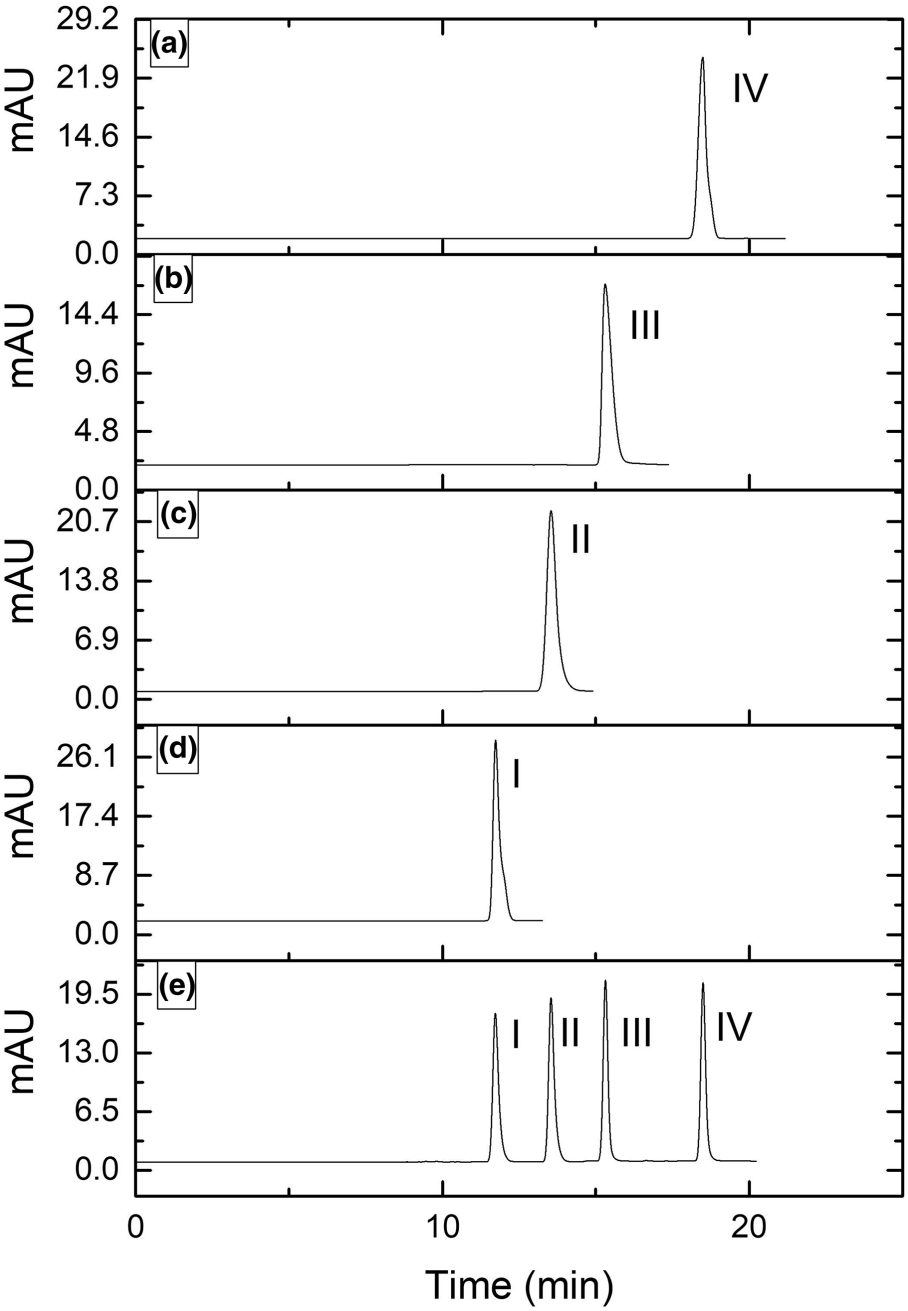
HPLC profile of different individual anthocyanins. Profile a–d are the solutions fractionated by a Sephadex LH‐20 column. (a) for cyanidin 3‐xyloside, (b) for cyanidin 3‐arabinoside, (c) for cyanidin 3‐glucoside, and (d) for cyanidin 3‐galactoside, (e) is the standard individual anthocyanins as the reference compounds to identify the different fractions, I for cyanidin 3‐galactoside, II for cyanidin 3‐glucoside, III for cyanidin 3‐arabinoside, and IV for cyanidin 3‐xyloside.

Two‐thousand‐gram *A. melanocarpa* (Michx.) Ell. was used to extract and purify the crude anthocyanins and its cyanidin glycosides. The results are shown in Table 5 and Figure 2. For crude extract of anthocyanins (CEA), there were not only anthocyanins but also water‐soluble compounds such as protein, sugar, and vitamin. Thus, more compounds were obtained at this stage. Followed by the absorbance of anthocyanins using Amberlite XAD7HP, most of the water‐soluble compounds have been washed off and methanol was used to get the high‐purity anthocyanin complex. There were mainly four cyanidin glycosides found in *A. melanocarpa* (Michx.) Ell. such as cyanidin 3‐galactoside, cyanidin 3‐arabinoside, cyanidin 3‐xyloside, and cyanidin 3‐glucoside, which were purified and showed the same migration time compared with the standard individual anthocyanins (Figure 2). Their chromatographic spectrum was in agreement with previous studies (Bräunlich et al., 2013). Unlike other berries, *A. melanocarpa* (Michx.) Ell. anthocyanin is very simple, consisting almost exclusively of the cyanidin glycoside as shown in our results in Table 5. However, Wu et al. (2004) found that there were minor amounts of pelargonidin‐3‐arabinoside and traces of pelargonidin‐3‐galactoside in *A. melanocarpa* (Michx.) Ell. (Wu et al., 2004).

**TABLE 5:** Extracts and fractions of anthocyanins from *A. melanocarpa* (Michx.) Ell.

Samples	Yields (mg)	Contents (mg/100 g FW)
Crude extract of anthocyanins (CEA)	67543.0 ± 505	3377.2
Anthocyanin‐enriched extract (AEE)	9442.3 ± 30	472.1
Cyanidin 3‐galactoside	5705.0 ± 23	285.3
Cyanidin 3‐arabinoside	2091.5 ± 11	104.6
Cyanidin 3‐xyloside	507.1 ± 5	25.4
Cyanidin 3‐glucoside	92.5 ± 2	4.6
Total anthocyanins content calculated by individual cyanin glycoside fractions	–	419.9

Cyanidin 3‐galactoside was the highest content individual anthocyanin with 60.42% content in AEE, followed by cyanidin 3‐arabinoside with 22.15% content, cyanidin 3‐xyloside with 5.37% content, and cyanidin 3‐glucoside with 0.98% content. Also, there still was an 11.08% loss of AEE which was ascribed to the impurity substances in AEE, and elute and recycle loss during the fractionation process. Cyanidin 3‐galactoside and cyanidin 3‐arabinoside are the predominant representatives of 82.57%, although this was lower than Oszmiański and Wojdylo (2005) reported with a cumulative content of >90% in the berries (Oszmiański & Wojdylo, 2005).

The total anthocyanins content calculated by individual cyanin glycoside fractions was 419.9 mg/100 g FW, which was lower than that determined by pH differentiation method as shown in Table 5. This error may have come from the recycle loss of the fractionation method, and impurities were calculated as anthocyanins during pH differentiation determination.

### 3.3 Extraction, fractionation, and characterization of proanthocyanidins

**FIGURE 3:**
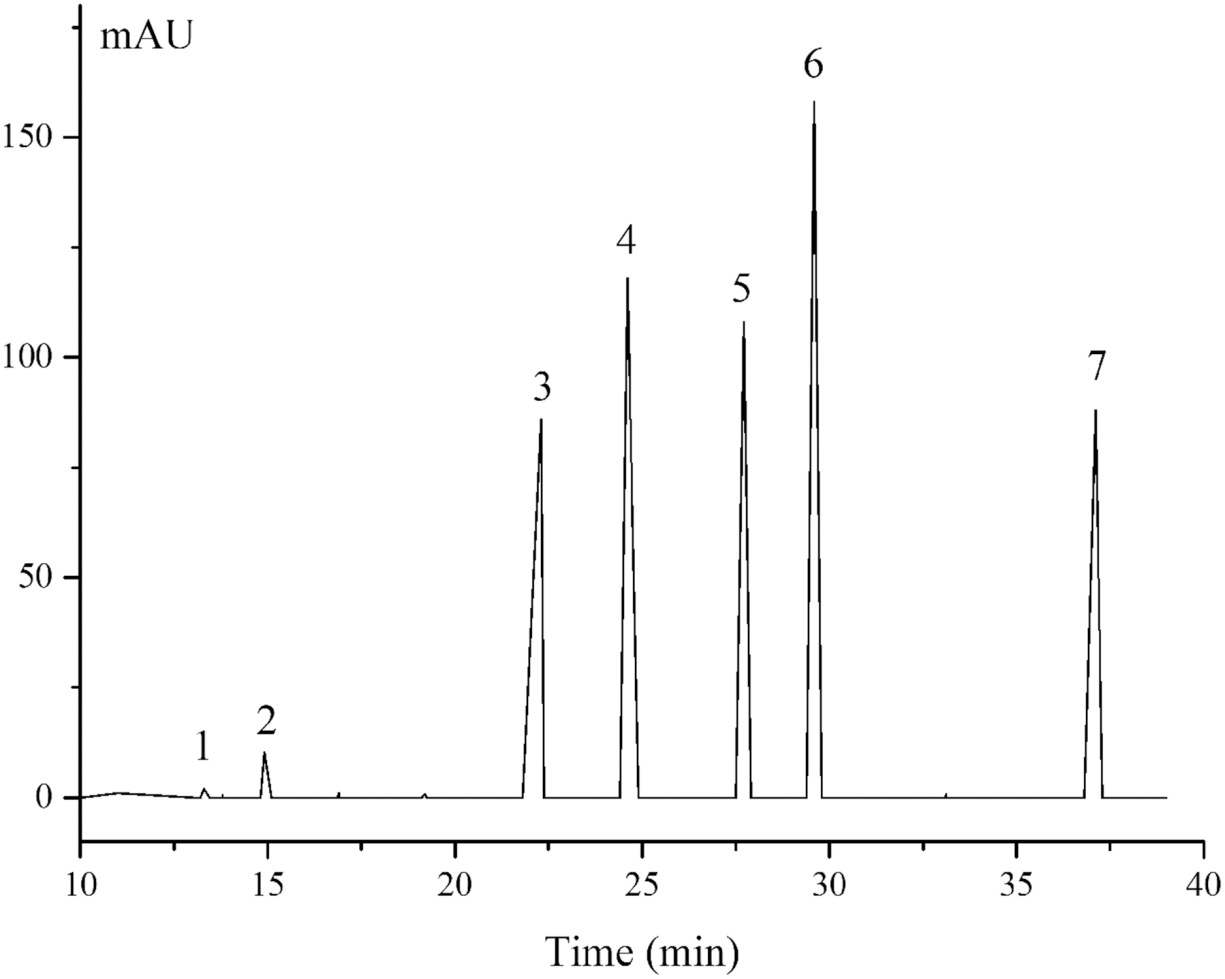
HPLC analysis of thiolysis solution of PP‐50 detected at 280 nm.

Two thousand gram of *A. melanocarpa* (Michx.) Ell. was used to fractionate the proanthocyanidins and found that polymeric proanthocyanidins constitute the major class of phenolics in *A. melanocarpa* (Michx.) Ell. Proanthocyanidins of *A. melanocarpa* (Michx.) Ell. are mainly composed of (−) epicatechin with trace amounts of catechin (Figure 3 and Table 6). The chain extension units and chain‐terminating units in proanthocyanidins were predominant as (−) epicatechin trace catechin (Figure 3), and the small peak 1 may be catechin. The mDP value has been calculated according to the thiolysis of proanthocyanidins as indicated in Table 7.

**TABLE 6:** MS spectrum of thiolysis solution of PP‐50.

Peak number	RT (min)	[M‐H]^−^(m/z)	Compounds
1	13.30	289	Catechin
2	14.91	289	(−) epicatechin
3	22.30	413	heterocyclic ring cleavage products of epicatechin
4	24.60	412	Epicatechin benzyl thioether
5	27.71	535	Heterocyclic ring cleavage products of epicatechin benzyl thioether
6	29.60	535	Heterocyclic ring cleavage products of epicatechin benzyl thioether
7	37.11	123	Benzyl mercaptan

**TABLE 7:** Extracts and fractions of proanthocyanidins with different IC_50_ from *A. melanocarpa* (Michx.) Ell.

	Yield (mg)	Content (mg/100 g FW)	mDP	IC_50_ values (μg/mL)		
				α‐Amylase (Worsztynowicz et al., 2014)	α‐Glucosidase (Bräunlich et al., 2013)	Lipase
Crude Proanthocyanidins extract (CPE)	29678.6 ± 30.5	1483.9 ± 1.5	49.3 ± 3.8	1.16 ± 0.12	0.81 ± 0.15	1.79 ± 0.06
Crude oligomer fraction from CPE (COF)	6618.5 ± 36.6	330.9 ± 1.8	1.8 ± 0.5	–	–	–
Monomers	446.1 ± 26.0	22.3 ± 1.3	–	–	–	–
Oligomers without monomers (OWM)	6072.0 ± 22.1	303.6 ± 3.1	4.2 ± 0.9	4.32 ± 0.21	3.99 ± 0.42	5.03 ± 0.55
Crude polymerized proanthocyanidins (CPP)	22906.4 ± 28.9	1145.3 ± 1.4	56.9 ± 2.5	1.01 ± 0.06	0.75 ± 0.03	1.61 ± 0.02
CPP‐50	6546.8 ± 24.5	327.3 ± 1.2	78.9 ± 4.1	0.23 ± 0.01	0.15 ± 0.06	0.31 ± 0.13
CPP‐60	8248.0 ± 21.6	412.4 ± 1.1	66.1 ± 1.2	0.83 ± 0.05	0.72 ± 0.02	1.57 ± 0.06
CPP‐70	3956.0 ± 30.1	197.8 ± 1.5	36.8 ± 3.9	1.39 ± 0.02	0.98 ± 0.03	2.05 ± 0.20
CPP‐75	1814.2 ± 27.3	90.7 ± 1.4	25.2 ± 1.3	2.46 ± 0.05	1.41 ± 0.04	3.03 ± 0.11
CPP‐L	2052.1 ± 24.4	102.6 ± 1.2	10.2 ± 2.6	3.01 ± 0.01	2.56 ± 0.05	4.56 ± 0.31

Abbreviation: – not determined.

The crude proanthocyanidins extract (CPE) was fractionated into six parts with different mDP. The mDP of the CPE was 49.3 ± 3.8, almost as reported by Skupien and Oszmianski (2007). After fractionation of the CPE, the proanthocyanidins were nicely fractionated with different mDP from 4.2 to 78.9, which will be ready for the studying of the enzyme inhibition with different mDP of proanthocyanidins as indicated in Table 7. The sum of OWM and CPP contents were the total proanthocyanidins, which was a little bit high than Table 4 indicated which was caused by different determination methods.

The mDP fractions of *A. melanocarpa* (Michx.) Ell. ranged from 1.8 ± 0.5 of COF to 78.9 ± 4.1 of CPP‐50 and that of the whole *A. melanocarpa* (Michx.) Ell. was 49.3 ± 3.8. The mDP of proanthocyanidins from *A. melanocarpa* (Michx.) Ell. was 12–70 in most of the reports and also has reported an exceptionally high mDP > 100 (Skupien & Oszmianski, 2007), differences may be caused by the different processing, storage, cultivation, determination methods, etc. Labarbe et al. (1999) also found that the proanthocyanidins from the seed and skin of grape also can be fractionated with different mDP, ranging increasingly from 4.7 to 17.4 in seed (8.1 for total extract) and from 9.3 to 73.8 in skin (34.9 for total extract). The monomer content of proanthocyanidins was the highest compared with several reports with content of 5.89 mg ECE/100 g FW (Dudonné et al., 2015), 5.17 mg/100 g FW (Wu et al., 2004), 0.01–0.02 μg CE/g DM (Taheri et al., 2013).

The composition of different mDP of proanthocyanidins was as follows: monomers (1.51%), oligomer (mDP of 4.2 ± 0.9, 20.57%), CPP‐50 (mDP of 78.9 ± 4.1, 22.17%), CPP‐60 (mDP of 66.1 ± 1.2, 27.94%), CPP‐70 (mDP of 36.8 ± 3.9, 36.8%), CPP‐75 (mDP of 25.2 ± 1.3, 6.14%), CPP‐L (mDP of 10.2 ± 2.6, 6.95%), and there were recycling loss of 0.34%. Wu et al. (2004) reported that the proanthocyanidins composition in *Aronia* is as follows: monomers (0.78%), dimers (1.88%), trimers (1.55%), 4–6‐mers (6.07%), 7–10‐mers (7.96%), and >10‐mers (81.72%) (Wu et al., 2004). This confirmed the conclusion that there was much more tannin in *A. melanocarpa* (Michx.) Ell., although there were 22.08% of monomer and oligomer higher than Wu et al. (2004) studies and 77.58% of mDP > 10 proanthocyanidins which are lower than that of other studies (Wu et al., 2004).

Proanthocyanidins' tendency to link with proteins is responsible for the astringency of *A. melanocarpa* (Michx.) Ell. (Tomar et al., 2022). Also, their pharmaceutical effects are interesting because they act beneficially on the circulatory system and are efficient free radical scavengers (Rigaud et al., 1993). Several studies found that proanthocyanidins with high mDP are more potent antioxidants than simple phenolics, and the increasing mDP may enhance the antioxidant power and lipase inhibition of proanthocyanidins (condensed tannins) (Sosnowska et al., 2016), which will influence its organoleptic or pharmacological properties. However, the high mDP feature should limit their absorption through the gut barrier; oligomers larger than trimers are unlikely to be absorbed in the small intestine in their native forms (Denev et al., 2012).

These studies established a rapid method to fractionate different mDP proanthocyanidins by different solvents only, which avoid the poor resolution and irreversible adsorption during chromatographic separations of the highly polymerized fraction (Labarbe et al., 1999).

### 3.4 Inhibition of the enzyme

**FIGURE 4:**
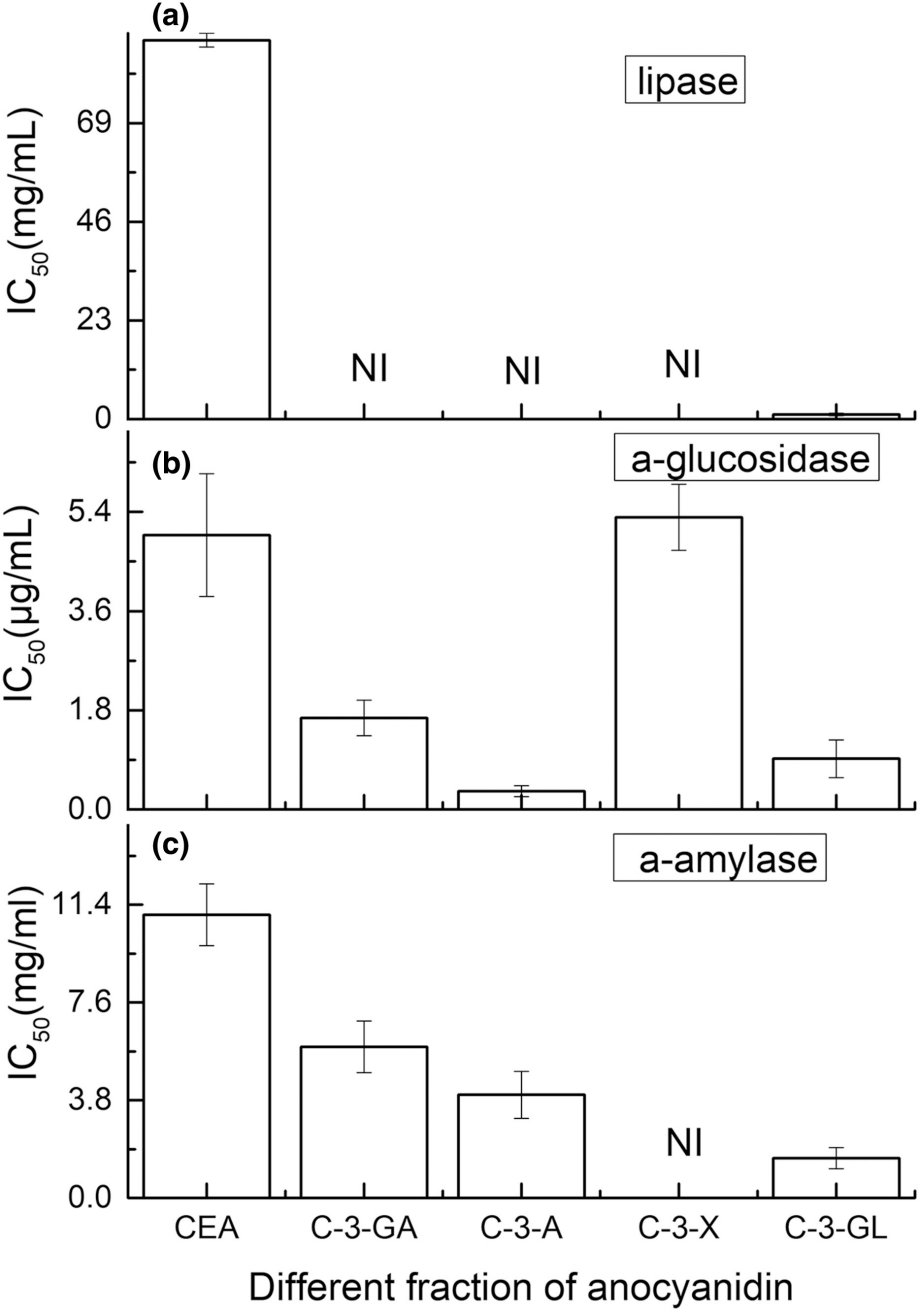
IC_50_ of enzyme inhibition effects by different individual cyanidin glycosides. NI indicates no inhibition.

The effects of crude extractions of anthocyanins and proanthocyanidins, the individual cyanin glycoside and proanthocyanidins with different mDP on the α‐amylase, α‐glucosidase and lipase are studied to identify the fractions of anthocyanins and proanthocyanidins that are responsible for the anti‐diabetes and obesity functions in *A. melanocarpa* (Michx.) Ell., The results were showed in Figure 4 and Table 7.

Among the individual cyanidin glycoside and crude anthocyanins extracts, cyanidin 3‐glucoside showed the strongest inhibition effects on α‐amylase and lipase and cyanidin 3‐arabinoside showed the strongest inhibition effect on α‐glucosidase, while cyanidin 3‐xyloside has no effect for the inhibition of α‐amylase. And cyanidin 3‐galactoside, cyanidin 3‐arabinoside, and cyanidin 3‐xyloside have no inhibition effects for the lipase. Cyanidin 3‐arabinoside showed the strongest inhibition effect on the three enzymes of the anthocyanins studied. These results showed the same tendency as indicated in Table 8. Considering the data obtained from our investigations (Figure 4) and the reports in Table 8, cyanidin glycosides may be one of the reasons for the diabetes treatment effect of *A. melanocarpa* (Michx.) Ell. (Chrubasik et al., 2010), and studies suggested that intake of cyanidin and glycosides‐enriched plant foods together with acarbose may lead to the development of a novel combined therapy in type 2 diabetic patients (Akkarachiyasit et al., 2010).

**TABLE 8:** IC_50_ values of individual cyanidin glycosides in different references.

Individual cyanidin glycoside/mDP	IC_50_ values		
	α‐Amylase	α‐Glucosidase	Lipase
Cyanidin 3‐galactoside	>1.00 mM (Akkarachiyasit et al., 2010) 5.16 ± 0.06 mg/mL (Worsztynowicz et al., 2014)	224.5 ± 22.45 μg/mL for sucrose (Adisakwattana et al., 2009) 1.54 ± 0.1 μg/mL (Bräunlich et al., 2013)	nf (Worsztynowicz et al., 2014)
Cyanidin 3‐arabinoside	3.98 ± 0.10 mg/mL (Worsztynowicz et al., 2014)	0.37 ± 0.08 μg/mL (Bräunlich et al., 2013)	nf (Worsztynowicz et al., 2014)
Cyanidin 3‐xyloside	nf (Worsztynowicz et al., 2014)	5.5 ± 1.6 μg/mL (Bräunlich et al., 2013)	nf (Worsztynowicz et al., 2014)
Cyanidin 3‐glucoside	134.7 ± 4.49 μg/mL (Akkarachiyasit et al., 2010) 1.74 ± 0.04 mg/mL (Worsztynowicz et al., 2014)	435.53 ± 22.45 μg/mL for sucrose (Akkarachiyasit et al., 2010); 0.87 ± 0.2 μg/mL (Bräunlich et al., 2013)	84.54 ± 2.94 μg/mL (Vijayaraj et al., 2019) 1.17 ± 0.04 mg/mL (Worsztynowicz et al., 2014)
Proanthocyanidins with different mDP (Data shown as mDP‐IC_50_)	3.3‐(0.075 ± 0.003) mg/mL (Fu et al., 2015) (11.8 ± 0.1)‐1.7 μg/mL (Kato et al., 2017) 32.6–2.9 μg/mL (Kato, 2019) 9.0–4.2 μg/mL (Kato, 2019) (4–8)‐38 μg/mL (Kato, 2019) (7.54 ± 0.22)–(1022.84 ± 31.50) μg/mL (Li et al., 2015) 11.69 ± (0.23–541.26) ± 20.30 μg/mL (Li et al., 2015)	(3.2–14.0)–(1–0.015) μg/mL (Hsu et al., 2018) (7.54 ± 0.22)‐(4.08 ± 0.56) μg/mL (Li et al., 2015) (11.69 ± 0.23)‐(3.12 ± 0.79) μg/mL (Li et al., 2015) (7.3 ± 0.1)‐(0.037 ± 0.001) mg/mL (Wang et al., 2019)	3.8‐(3.88 ± 0.35) μg/mL (Ci et al., 2018) 9.6‐(1.84 ± 0.46) μg/mL (Ci et al., 2018)
One more study for mDP and IC_50_ with figure is Zhou et al. (2018)			

Abbreviation: nf, not found.

**FIGURE 5:**
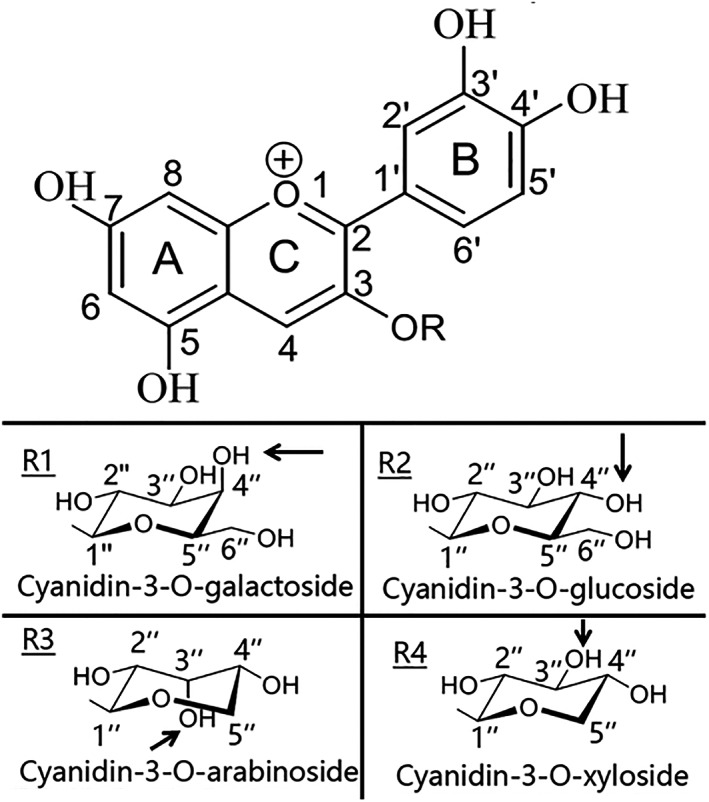
Different molecule structures of cyanidin glycosides.

This was the first study to investigate all the individual cyanidins and glycoside interactions on the enzyme inhibition effects from *A. melanocarpa* (Michx.) Ell. It indicated that the structural difference between glycoside at the 3‐O‐position of cyanidin was an important factor for modulating the inhibition of the three enzymes. The glycosides (glucose and galactose, arabinose, and xylose) of the individual anthocyanins have the same formulas but different structural formulas with different positions of the hydroxyl (‐OH) group on C‐4 for cyanidin 3‐galactoside and cyanidin 3‐glucoside and c‐3 for cyanidin 3‐arabinoside and cyanidin 3‐xyloside as indicated in Figure 5 with arrows, which suggests that the structural difference in the sugar at the 3‐O‐position may be an important factor for modulating the inhibition of the enzyme studied (Akkarachiyasit et al., 2010). Also, the B‐ring increases the hydrophobic characteristics of the compounds, thus enhancing higher affinity toward the enzyme (Vijayaraj et al., 2019). However, it is still difficult to tell the reasons why these other three anthocyanins have no inhibition effects on lipase, and more studies such as the interaction between the enzyme and anthocyanins molecules are needed.

**FIGURE 6:**
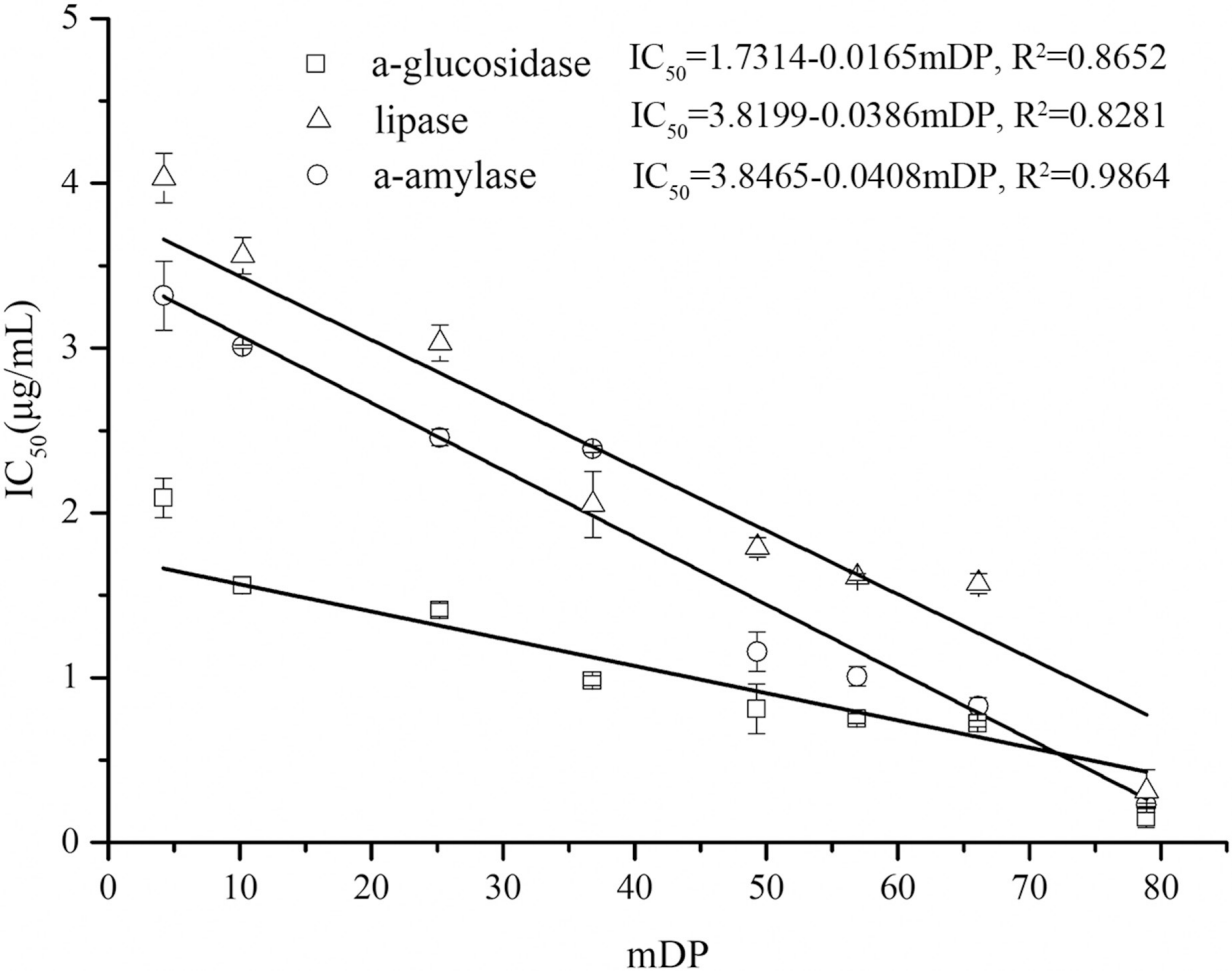
Correlation of the mDP and IC_50_ of proanthocyanidins.

Table 8 also indicated the inhibition effects of the proanthocyanidins with different mDP, which are normally distributed. According to the results, inhibition of α‐glucosidase is more specific than inhibition of lipase and α‐amylase. And PP‐50 showed the strongest inhibition effects on the three enzymes. However, the effects of the three enzymes showed high negative correlations between the mDP and IC_50_ (Figure 6). Thus, it indicated that the enzyme inhibition effect of proanthocyanidins from *A. melanocarpa* (Michx.) Ell. was mDP correlated and the presence of epicatechin as the extension unit may play an important role (Kato et al., 2017). The main inhibitory mechanism of proanthocyanidins on the enzyme studied may be due to the insertion of proanthocyanidins into the pocket of the enzyme altering the catalytic configuration of the active site in a manner, thus reducing substrate‐binding affinity (Wei et al., 2017). All the proanthocyanidins fractions with different mDP showed strongest inhibition effects than anthocyanins individuals on α‐amylase. And CPP‐50 showed stronger α‐glucosidase and lipase inhibition than that of all the anthocyanins. This may be ascribed to the most effective protein‐precipitating effect of large molecules of proanthocyanidins (Hofmann et al., 2006), which is of particular importance, as bioavailability is not needed for proanthocyanidins of any size to exert activity by inhibiting the digestion of lipids or carbohydrates in the gastrointestinal lumen (Neilson et al., 2016).

## 4 CONCLUSIONS

*A. melanocarpa* (Michx.) Ell. has been transplanted to China for about 12 years, and it has only been allowed to be sold as food in China for not more than 4 years. Therefore, the functional components and functions of the fruit planted in China still need further study. The results showed that the content, composition, and enzyme inhibition activity of anthocyanin and proanthocyanidins of *A. melanocarpa* (Michx.) Ell. from China were similar to those in the United States and Europe. Anthocyanin and proanthocyanidins inhibited the activity of the digestion enzyme, which is one of the important physiological functions of *A. melanocarpa* (Michx.) Ell. However, the interaction and mechanism between anthocyanin and proanthocyanidins and enzymes need to be further studied for the development of functional foods.

## AUTHOR CONTRIBUTIONS

**Limei Chen:** Data curation (equal); formal analysis (equal); investigation (equal); methodology (equal); project administration (equal); software (equal); validation (equal); visualization (equal); writing – original draft (equal); writing – review and editing (equal). **Wuxi Chen:** Data curation (equal); methodology (equal); validation (equal); visualization (equal); writing – original draft (equal); writing – review and editing (equal). **Demao Li:** Formal analysis (equal); funding acquisition (equal); investigation (equal); software (equal); writing – review and editing (equal). **Xiumin Liu:** Conceptualization (equal); funding acquisition (equal); project administration (equal); resources (equal); supervision (equal); writing – original draft (equal); writing – review and editing (equal).

## FUNDING INFORMATION

This research was funded by the National Key R&D Program of the Ministry of Science and Technology of China (2018YFA0902200).

## CONFLICT OF INTEREST STATEMENT

The authors declare no conflict of interest. The funders had no role in the design of the study; in the collection, analyses, or interpretation of data; in the writing of the manuscript; or in the decision to publish the results.

## Data Availability

The data that support the findings of this study are available on request from the corresponding author. The data are not publicly available due to privacy or ethical restrictions.
